# Disrupted-in-schizophrenia 1 enhances the quality of circadian rhythm by stabilizing BMAL1

**DOI:** 10.1038/s41398-021-01212-1

**Published:** 2021-02-04

**Authors:** Su Been Lee, Jihyun Park, Yongdo Kwak, Young-Un Park, Truong Thi My Nhung, Bo Kyoung Suh, Youngsik Woo, Yeongjun Suh, Eunbyul Cho, Sehyung Cho, Sang Ki Park

**Affiliations:** 1 Department of Life Sciences, Pohang University of Science and Technology, Pohang, Republic of Korea; 2 Department of Physiology, College of Medicine, Kyung Hee University, Seoul, Republic of Korea; 3 Present Address: SK biopharmaceuticals Ltd, Seongnam-Si, Republic of Korea; 4 Present Address: Department of Pathology, College of Medicine, Hanyang University, Seoul, Korea

**Keywords:** Physiology, Molecular neuroscience

## Abstract

Disrupted-in-schizophrenia 1 (DISC1) is a scaffold protein that has been implicated in multiple mental disorders. DISC1 is known to regulate neuronal proliferation, signaling, and intracellular calcium homeostasis, as well as neurodevelopment. Although DISC1 was linked to sleep-associated behaviors, whether DISC1 functions in the circadian rhythm has not been determined yet. In this work, we revealed that *Disc1* expression exhibits daily oscillating pattern and is regulated by binding of circadian locomotor output cycles kaput (CLOCK) and Brain and muscle Arnt-like protein-1 (BMAL1) heterodimer to E-box sequences in its promoter. Interestingly, *Disc1* deficiency increases the ubiquitination of BMAL1 and de-stabilizes it, thereby reducing its protein levels. DISC1 inhibits the activity of GSK3β, which promotes BMAL1 ubiquitination, suggesting that DISC1 regulates BMAL1 stability by inhibiting its ubiquitination. Moreover, *Disc1*-deficient cells and mice show reduced expression of other circadian genes. Finally, *Disc1*-LI (*Disc1* knockout) mice exhibit damped circadian physiology and behaviors. Collectively, these findings demonstrate that the oscillation of *DISC1* expression is under the control of CLOCK and BMAL1, and that DISC1 contributes to the core circadian system by regulating BMAL1 stability.

## Introduction

Anticipating the time of day is crucial for organisms that live under recurrent sunlight. The molecular circadian clock system maintains the daily cycle of organisms^1–5^. Transcription–translation feedback loop (TTFL) of circadian genes ensures this cycle, which keeps the circadian clock ticking even without external time cues^5^. In the mammalian TTFL system, the main component is the heterodimer consisting of Circadian locomotor output cycles kaput (CLOCK) and Brain and muscle Arnt-like protein-1 (BMAL1), which binds to E-box sequences of target genes for transcriptional enhancement^6,7^. In this way, the CLOCK/BMAL1 heterodimer upregulates core clock genes, including Period (*PERs*), Cryptochrome (*CRYs*), and Nuclear receptor subfamily 1 (*Nr1d1*)^5^. In turn, the action of CLOCK/BMAL1 heterodimer is negatively regulated by PERs and CRYs, thus completing the TTFL^8,9^.

Timed regulation of circadian proteins is essential to the proper working of the circadian timing system. To this end, the degradation of the molecular circadian components is vital. Newly synthesized circadian proteins must be degraded at the right time for the accurate pacing of the circadian clock. Indeed, several circadian components, including BMAL1, PERs, CRYs, and REV-ERBs, are regulated by degradation pathways^10^. Glycogen synthase kinase 3β (GSK3β) phosphorylates BMAL1, thereby enhancing its ubiquitination and degradation^11^. Moreover, casein kinases Iδ/ε (CKIδ/ε) phosphorylate PERs leading to their degradation^10^. Chemical inhibition of CKI augments the stability of PERs and induces a more extended circadian period. F-box and leucine-rich repeat protein 3 (FBXL3), an E3 ubiquitin ligase, mediates ubiquitination and subsequent degradation of CRYs^12^, and FBXL3-mutated mice also exhibit longer circadian period^13^. REV-ERBα is phosphorylated by cyclin-dependent kinase 1 (CDK1) and recognized for ubiquitination by F-box and WD repeat domain containing 7 (FBXW7)^14^. The loss-of-function of FBXW7 results in a damped circadian amplitude, indicating that the degradation of REV-ERBα is essential for the circadian amplitude. Altogether, these studies indicate that proper degradation of circadian proteins is a critical factor to maintain circadian period and amplitude.

Disrupted-in-schizophrenia 1 (*DISC1*) was first reported as a responsible gene for prevalent psychiatric conditions, including schizophrenia, in a Scottish pedigree^15^. Although DISC1 as a genetic risk factor for schizophrenia has more to be elucidated, DISC1 plays significant molecular roles, including functions in early brain development, which are important to the molecular basis of psychiatric disorders^16^. DISC1 is a scaffold protein with a large number of interacting partners that perform various functions in the nervous system, such as neuronal migration, neurite outgrowth, spine regulation, and synapse maintenance^17–20^. Interestingly, DISC1 is associated with sleep-related phenotypes. Expression of human DISC1 in fruit flies alters their sleep homeostasis^21^. Furthermore, a mouse model of DISC1 gain-of-function shows increased wakefulness and decreased REM and NREM sleep^22^. A close relationship between sleep and the circadian clock has been proposed^23^; however, potential direct involvement of DISC1 in the mammalian circadian system has not been explored yet. In this study, we investigated the relationship between DISC1 and the circadian system and proposed a modulatory role of DISC1 in the mammalian molecular clock.

## Materials and methods

### Animals

All animal-related experiments were approved by Pohang University of Science and Technology Institutional Animal Care and Use Committee (POSTECH-2019-0024 and POSTECH-2019-0025), and animal experiments were conducted with approved guidelines. Wild type C57BL/6J mice were purchased from Hyochang Science (Daegu, South Korea). *Disc1* knockout mouse, named as *Disc1* locus impairment mouse (*Disc1*-LI mouse, C57BL/6 J backgrounded), was previously described^24^ and a kind gift from Dr. Akira Sawa, Johns Hopkins University School of Medicine. In *Disc1*-LI mouse, exons from 1 to 3 of *Disc1* were replaced to Neomycin resistance cassette by homologous recombination^24,25^. Male wild type (C57BL/6J) mice and *Disc1*-LI mice were housed under 12-h light 12-h dark conditions with *ad libitum* access to food and water. Male mice aged 3 to 4 months were employed for circadian experiments.

### Cell culture and transfection

HEK293 cell line, NIH3T3 cell line, and Mouse embryonic fibroblasts (MEFs) were maintained in 5% CO_2_ incubator at 37 °C with DMEM high glucose (Welgene, LM 001-05) containing 10% fetal bovine serum (Merck, ES009B-KC) and 1% penicillin/streptomycin (HyClone, SV30010). Cells were transfected with Polyethylenimine (PEI, Polysciences, Inc., 23966-2) as previously described^26^. Briefly, plasmids were mixed with PEI (1 mg/ml, pH 7.0) in opti-MEM (Gibco, 31985-070) and added to cells after 15 min of incubation.

### Antibodies

For antibodies, rabbit anti-DISC1 (Millipore, ABN-425) was purchased from Millipore; rabbit anti-BMAL1 (Abcam, ab93806) from Abcam; mouse anti-alpha-tubulin (Proteintech, 66031-1-lg) from Proteintech; rabbit anti-HA (Bethyl, A190-108A) from Bethyl; rabbit anti-GFP (Invitrogen, A11122) from Invitrogen; rabbit anti-Flag (ThermoFisher, PA1-984B) from ThermoFisher; mouse anti-Flag (Sigma, F1804) from Sigma; mouse anti-c-Myc (Santa Cruz, sc-40) from Santa Cruz; GSK3β p-Y216 (Invitrogen, 44-604G) from Invitrogen; mouse anti-GSK3β (Cell Signaling, 9832S) from Cell Signaling. Antibodies were utilized as the first antibody of western blot or precipitating antibody of immunoprecipitation.

### Plasmids

Human *DISC1* promoter (-982 to +47 relative to TSS^27^) was cloned in pGL3-basic (Promega), whose luciferase was modified as destabilized with PEST sequence^28^. For distal, middle, and proximal promoter regions of *DISC1*, −982 to −624, −623 to −324, and −323 to +47 relative to TSS respectively, were cloned in pGL3-basic with destabilized luciferase. PCR and DpnI-based mutation method was utilized to mutate E-box sequences of *DISC1* promoter. Primers were used for the PCR; for mutant E-box (−718 relative to TSS): 5′-TCTATGACCGTACTCTCCTTC-3′ and 5′-GAAGGAGAGTACGGTCATAGA-3′; for mutant E-box (−668 relative to TSS): 5′-AGAGCTACTGTATAGCCCTTC-3′ and 5′-GAAGGGCTATACAGTAGCTCT-3′. Human CLOCK cDNA was cloned in mRFP-C1 with myc tagging at C-terminal. Human BMAL1 cDNA was cloned in pEGFP-C1 (Clontech) and pcDNA3.1 myc-His C (Invitrogen). HA-hDISC1 was a kind gift from Dr. Akira Sawa, Johns Hopkins University School of Medicine, and sub-cloned in pFLAG-CMV2 (Sigma). Human GSK3β cDNA was cloned in pcDNA3.1 myc-His C (Invitrogen). HA-Ubiquitin was a kind gift from Dr. Chin Ha Chung, Seoul National University, Seoul, Korea. pLL3.7 vector was utilized for shRNA knockdown experiments. Target sequence for control shRNA, 5′-ACTACCGTTGTATAGGTG-3′, was previously described^29^; 5′-GCAGGAGGTCAGCAAGGCCTTG-3′ for human DISC1 shRNA was previously described^30^.

### Western blot

For western blot, cells or brain samples were lysed in Nonidet P-40 buffer (50 mM Tris-Cl, pH 8.0, 150 mM NaCl, 1% NP40, 5 mM EDTA, 1X protease inhibitor cocktail). Samples were sonicated and centrifuged at 13,000 rpm, 4 °C for 10 min to remove debris. Acquired supernatant was mixed with SDS sampling buffer and denatured at 95 °C for 10 min. Denatured samples were separated with SDS-PAGE gel electrophoresis and transferred to PVDF membrane (Immobilon-PSQ PVDF Membrane, Millipore, ISEQ00010). Membranes were blocked with 5% skim milk and incubated with each appropriate first antibody for overnight at 4 °C, then washed 3 times with TBS-T buffer (20 mM Tris-Cl, pH 7.6, 137.5 mM NaCl, 0.25% Tween 20). Further, membranes were incubated with Horseradish peroxidase (HRP) conjugated second antibody for 1 h, followed by 3 times washing with TBS-T buffer. ECL solution (Bio-rad, 1705061) was applied to membranes, and the band signals were detected by chemiluminescent detector (Azure, c280).

### Quantitative RT-PCR

Tri-solution (Bio Science Technology, TS200-001) was used for extracting RNA from cells or brain samples. The RNA concentration was measured by Nanodrop 2000 (Thermo), and 1 μg of RNA was subjected to further experiments. ImProm-II™ Reverse Transcription System (Promega, A3800) and poly T primer were used for reverse transcript of RNA samples. FastStart Universal SYBR Green Master (Rox) (Roche, 04913850001) and StepOnePlus Real-Time PCR System (Applied Biosystems) were utilized to conduct qPCR according to manufacturer’s instructions with primers for each gene:

Period1 F: 5′-GCCAAGAAAGATCCGTCGTCAG-3′

Period1 R: 5′-GGGCTTCTTGTCTCCCACATGGAC-3′ (ref. ^31^)

Period2 F: 5′-GCTTCTGGTCTGGACTGCAC-3′

Period2 R: 5′-GAGTGTCTGAGGGCTCGTTG-3′ (ref. ^32^)

Cryptochrome1 F: 5′-GTGGATCAGCTGGGAAGAAG-3′

Cryptochrome1 R: 5′-CACAGGGCAGTAGCAGTGAA-3′ (ref. ^33^)

Bmal1 F: 5′-GCAGTGCCACTGACTACCAAGA-3′

Bmal1 R: 5′-TCCTGGACATTGCATTGCAT-3′ (ref. ^34^)

Disc1 F: 5′-GAACAGCAGAAGGCTGGGC-3′

Disc1 R: 5′-GACCTTCCAACACTTCCATGC-3′ (ref. ^35^)

Tbp F: 5′-GGGAGAATCATGGACCAGAA-3′

Tbp R: 5′-CCGTAAGGCATCATTGGACT-3′ (ref. ^36^)

Gapdh F: 5′-CACTGAAGGGCATCTTGG-3′

Gapdh R: 5′-TTACTCCTTGGAGGCCATG-3′ (ref. ^37^)

*Gapdh* or TATA-Box Binding Protein (*TBP*) was utilized as a reference gene for qRT-PCR according to previous studies^38–42^.

### Mouse embryonic fibroblasts

Mouse embryonic fibroblasts (MEFs) were obtained from wild type C57BL/6J mouse and *Disc1*-LI C57BL/6J mouse line. Embryos with embryonic day 14.5 were used. Body part of embryos without red blood organs was washed with PBS (Welgene, ML 008-01) and minced with blades. Samples were dissociated further with trypsin-EDTA (HyClone, SH30042.02) and pipetting for 10 min. Complete media (DMEM with 10% FBS and 1% penicillin/streptomycin) was applied to block trypsin activity. Collected cells were centrifuged for 3 min at 1000 rpm and pellets were resuspended with complete media. To dissociate into single cells, cell strainer was applied. Dissociated cells were seeded into culture dishes and media change was conducted after 6 h.

### Circadian rhythm experiments

For cultured cell experiments, 1 μM of dexamethasone (Sigma, D4902-25MG) was applied to synchronize cells’ circadian phase. After 2 h, cells were changed to fresh complete media (DMEM with 10% FBS and 1% penicillin/streptomycin) and were harvested according to each time point. To obtain mouse brain samples for circadian experiments, mice were entrained to a 12-h light and 12-h dark cycle for 7 days and then to constant darkness to monitor internal circadian phenotypes. After the constant dark cycle had started, mice were kept for 1 day and sacrificed in the next day. 36 h after the constant dark cycle had started was considered circadian time 0. Mice were sacrificed under dim red light according to their circadian times and further dissection for brains was performed.

For long-term monitoring of circadian behavior in live animals, mice were surgically implanted with telemetric proves (E-mitter; Mini-Mitter, Bend) and kept in a light-proof Clean Animal Rack cabinet (Shin Biotech) equipped with an automatic light–dark schedule controller. The light intensity during the light phase was kept at 350–450 lux all over the cabinet. The temperature inside the cabinet was maintained to 23 ± 1 °C, and constant air ventilation was applied all the time. Prior to the actual monitoring, mice were fully entrained to a 12-h light and 12-h dark cycle for at least two weeks. For the next 10 days with the same light–dark cycle, wheel-running activity, body temperature, and home cage activity were continuously recorded at 6-min intervals using ER-4000 system. Data obtained during this period were used for the construction of light–dark profiles of behaviors. Then, mice were released to constant darkness (dark-dark cycle) for 10 days to determine the free-running period of circadian behaviors. For phase delay assay or phase advance assay, 30 min of light pulse was given to mice on circadian time 14 h or 20 h, respectively. Thereafter, the phase change was measured.

### Luciferase assay

Luciferase assay was conducted using Dual Luciferase Assay system (Promega, E1910), according to manufacturer’s instruction. Cells were cultured on 12-well plate and transfected with PEI. Prior to cell prep, cells were washed with PBS (Welgene, ML 008-01). 100 μl of passive lysis buffer (PLB) was applied to each well, and the plate was agitated for 10 min to detach cells from the plate. The lysate was collected and centrifuged for 30 s at 13,000 rpm. The 40 μl of supernatant was transferred to 96-well white plate (Nunc, 136101). 50 μl of luciferase assay buffer II was injected into a well of 96-well plate, and shaking was performed for 1 second. Thereafter, Firefly luciferase activity was measured for 10 s. 50 μl of Stop & Glo Buffer was injected into the same well of 96-well plate, and shaking was performed for 1 s. Thereafter, Renilla luciferase activity was measured for 10 s. Injection of substrates and luminescence detection were programmed and conducted by automatic injector and plate reader (TECAN, infinite M200 PRO).

### Immunoprecipitation and ubiquitination assay

Cultured cells, transfected with proper plasmids for each experiment, were lysed with Nonidet P-40 buffer (50 mM Tris-Cl, pH 8.0, 150 mM NaCl, 1% NP40, 5 mM EDTA, 1× protease inhibitor cocktail). Brain samples were homogenized in Nonidet P-40 buffer. Samples were sonicated and centrifuged at 13,000 rpm, 4 °C for 10 min. The supernatants were incubated with 500 ng of antibodies via rotation at 4 °C. Protein A beads (Roche, 05015979001) were washed three times with Nonidet P-40 buffer. Antibody incubated samples were added to washed beads and incubated further for 4 h at 4 °C with rotation. Beads, with bound proteins, were precipitated with 1 min of centrifugation at 2000 rpm, 4 °C. Beads were washed three times with Nonidet P-40 buffer and denatured with SDS sampling buffer at 95 °C for 10 min. Samples were further subjected to immunoblotting. For ubiquitination assay, cells had been transfected with plasmids, including HA-ubiquitin; thereafter, 10 mM of MG132 (Sigma, C2211-5MG) was treated for 5 h prior to the cell lysis. Further experimental procedure is identical to immunoprecipitation assay.

### Chromatin immunoprecipitation

Cultured cells were transfected with appropriate plasmids. Cells were treated with 0.75% of formaldehyde (Sigma, 252549) dropwise and incubated for 10 min with slow agitation. 125 mM of glycine was treated to stop cross-linking. After twice of PBS washing, cells were harvested with PBS and centrifuged for 5 min, 1000 × *g*, 4 °C. Cells were resuspended with ChIP lysis buffer (50 mM HEPES-KOH pH 7.5, 140 mM NaCl, 1 mM EDTA pH 8.0, 1% Triton X-100, 0.1% sodium deoxycholate, 0.1% SDS, 1× Protease Inhibitor) and incubated for 10 min on ice. Samples were sonicated for 20 min (30% amplitude, sonicate/rest for 3 s/3 s), and cell debris was removed with centrifuge for 10 min, 13,000 rpm, 4 °C. Samples were diluted with mRIPA buffer (150 mM NaCl, 50 mM Tris-HCl pH 7.5, 1% NP-40, 1% Triton X-100, 0.5% sodium deoxycholate, 5 mM EDTA). 50 μl of samples were kept as an input. 25 μg of total DNA samples were used each. Samples were incubated with Protein A beads (Roche, 05015979001) for 4 h at 4 °C with rotation to pre-clear non-specific bindings. Beads were removed by centrifuge for 1 min, 2,000 rpm, 4 °C. Antibody was treated to samples and incubated for 4 h at 4 °C with rotation. New Protein A beads were washed three times with mRIPA buffer, and beads were incubated with samples for overnight. Incubated beads were washed once with Low salt buffer (0.1% SDS, 1% Triton X-100, 2 mM EDTA, 20 mM Tris-HCl pH 8.0, 150 mM NaCl), once with High salt buffer (0.1% SDS, 1% Triton X-100, 2 mM EDTA, 20 mM Tris-HCl pH 8.0, 500 mM NaCl), once with LiCl buffer (0.25 M LiCl, 1% NP-40, 1% sodium deoxycholate, 1 mM EDTA, 10 mM Tris-HCl pH 8.0) by centrifuge for 1 min, 2000 rpm, 4 °C. Bead-attached samples were eluted from beads by incubation with 120 μl of Elution buffer (1% SDS, 100 mM NaHCO_3_) for 15 min at 30 °C. Beads were removed by centrifuge for 1 min, 2000 rpm. 4.8 μl of 5 M NaCl and 2 μl of 10 mg/ml RNase A (Thermo, EN0531) were added to samples and incubated for overnight at 65 °C with agitation. 4 μl of 10 mg/ml Proteinase K (Sigma, P2308) was added and incubated for 1 h at 60 °C with agitation. Further, DNA was acquired by PCR purification kit. For inputs, 50 μl of each input was mixed with 70 μl of Elution buffer and followed the same procedure with bead samples. Promoter targets were quantified with qPCR. For E-boxes of *DISC1* promoter, 5′-GGTTTTGTGCCAAGCCTCTG-3′/5′-AGCTCACCTCCAGGCTAGAA-3′ and 5′-GGAGGTGAGCTGCTTAAGGG-3′/5’-CCATGCAAGCTCCTAGGCAA-3′ primer sets were utilized for qPCR.

### Quantification and statistical analysis

Sample size was chosen based on previous studies. No statistical methods were used for sample size estimation. No randomization was used for animal studies. The investigators were not blinded to the group allocation during the animal experiments. ImageJ (NIH) was used for quantification of band intensity of western blot, and Prism 8 (GraphPad) was used for statistical analyses. Statistical significances were determined by unpaired *t*-test or paired *t*-test, two-tailed for comparisons of two groups. One-way ANOVA or two-way ANOVA was used for comparisons of multiple groups, followed by Tukey’s multiple comparison test or Sidak’s multiple comparison test, respectively. *P*-value less than 0.05 was considered a significant difference. If variances of the data were significantly different, we utilized a statistical test that does not assume homogenous variance; Welch’s ANOVA, unpaired *t*-test with Welch’s correction.

## Results

### DISC1 shows rhythmic expression relative to circadian time

**Fig. 1:**
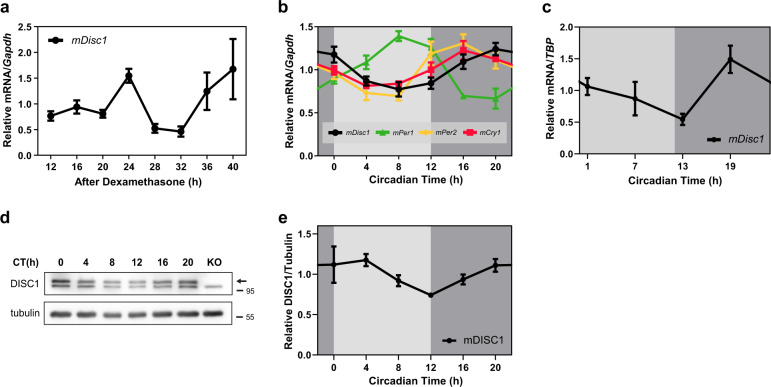
Expression of DISC1 exhibits an oscillating pattern. **a** mRNA expression of *Disc1* in NIH3T3 cells. Cells were treated with 1 μM of dexamethasone for 2 h to synchronize the circadian cycle. mRNA levels of *Disc1* were analyzed by qRT-PCR and compared to those of *Gapdh* (*n* = 5 for 12 h, *n* = 6 for other time points, biological replicates). The ratios of the relative mRNA levels at each time point to the mRNA levels averaged across all time points were presented. *p* = 0.0151, one-way ANOVA. **b** mRNA level of *Disc1* in hippocampus of wild type mice (*n* = 3, 3 mice for each time point) during the circadian time. Mice were entrained on a 12-h light/12-h dark cycle for a week; thereafter, mice were in the constant dark for a day and were sacrificed under dim red light according to their circadian time. mRNA levels of *Disc1* were analyzed using qRT-PCR and compared to those of *Gapdh*. To compare the expression patterns of circadian genes measured by qRT-PCR each other, the ratios of the relative mRNA levels at each time point to the mRNA levels averaged across all time points were presented. *p* = 0.0037 for *Disc1*, one-way ANOVA. **c** mRNA levels of *Disc1* in suprachiasmatic nucleus (SCN) of wild type mice (*n* = 3, 3 mice for 7 h; *n* = 4, 4 mice for other time points) during the circadian time. mRNA levels of *Disc1* were analyzed by qRT-PCR and compared to those of TATA-box binding protein (*TBP*). The ratios of the relative mRNA levels at each time point to the mRNA levels averaged across all time points were presented. *p* = 0.0166, one-way ANOVA. **d** DISC1 protein levels according to the circadian time in hippocampus were assessed by western blot. Wild type mice were entrained on a 12-h light/12-hr dark cycle for a week; thereafter, mice were in the constant dark condition and sacrificed under dim red light according to their circadian time. *Disc1* knockout (*Disc1*-LI mouse) sample was used as a negative control. Arrow indicates DISC1 bands. **e** Quantification of DISC1 protein level from (**d**) (*n* = 4 for each time point, biological replicates). Protein levels were quantified relative to tubulin. The ratios of the relative band intensity at each time point to the band intensities averaged across all time points were presented. *p* = 0.0042, Welch’s ANOVA. Data are means with SEM.

To elucidate whether *DISC1* is associated with the circadian system, we first examined whether *DISC1* itself has an oscillatory expression pattern as other circadian-related genes. *Disc1* mRNA expression was assessed in NIH3T3 cells after synchronization induced by dexamethasone treatment. The mRNA level of *Disc1* showed an oscillatory pattern that peaked at 24 and 40 h after synchronization (Fig. 1a). Subsequently, we examined this expression pattern on an organism level. We entrained mice to a 12-h light and 12-h dark cycle for a week, and then switched to constant darkness and sacrificed mice according to their circadian time. Remarkably, we revealed the oscillatory mRNA expression of *Disc1* in the hippocampus (Fig. 1b). *Disc1* mRNA peaked at circadian time 20 h (CT20), originally nighttime (~12–24 h) for mice. The mRNA oscillation of *Disc1* was also observed in the suprachiasmatic nucleus (SCN) (Fig. 1c). *Disc1* mRNA peak was CT19. When compared to other circadian genes in SCN, *Disc1* peak time is slower than *Per1* and *Per2*, and faster than *Rev-erbα*^43,44^. Moreover, DISC1 endogenous protein levels also oscillated (Fig. 1d, e). Taken together, *Disc1* expression shows an oscillatory pattern, with the expression level enhanced during late nighttime to early daytime.

### CLOCK and BMAL1 heterodimer regulates *DISC1* promoter

Next, we attempted to discover the factors that contribute to the oscillatory expression of *DISC1* within the molecular circadian system. As shown in Fig. 1b, the mRNA expression of *Disc1* increases in late nighttime and a little later, roughly similar to the expression pattern of *Cry1*. Since the expression of circadian genes, such as *Cry1*, *Cry2*, and *Per2*, is controlled by CLOCK/BMAL1 heterodimer^5^, we hypothesized that *Disc1* expression could be regulated with a comparable mechanism.

**Fig. 2:**
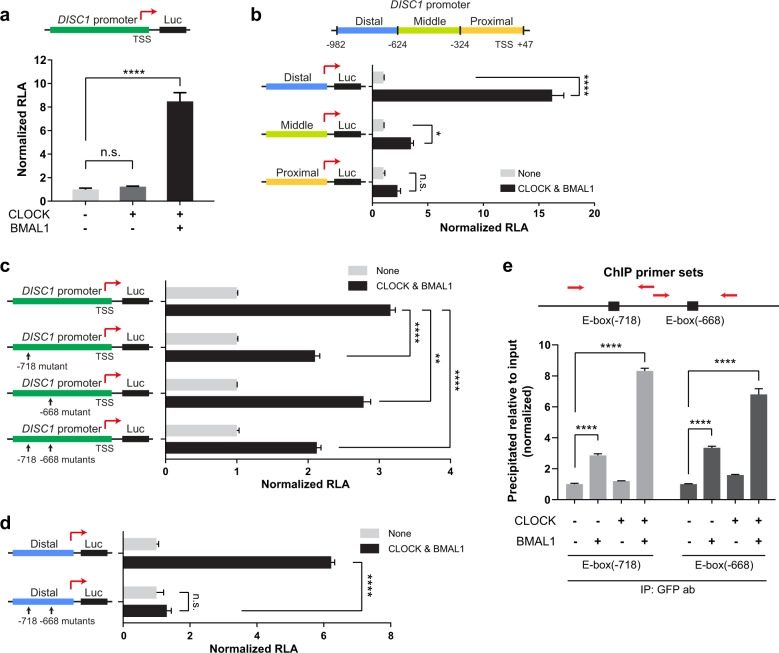
Promoter activity of *DISC1* is enhanced by CLOCK and BMAL1 through E-box sequences. **a** *DISC1* promoter was cloned into pGL3 vector to monitor promoter activity by luciferase expression. Luciferase assay was performed in HEK293 cells overexpressing CLOCK or together with BMAL1 (*n* = 3, biological replicates). **b** Luciferase assay with *DISC1* promoter sub-regions: distal part (−982 to −624 bp relative to TSS), middle part (−623 to −324 bp relative to TSS), and proximal part (−323 to +47 bp relative to TSS). Luciferase assay was performed in HEK293 cells overexpressing CLOCK and BMAL1 (*n* = 3, biological replicates). Each group was normalized to the respective control (empty vectors were transfected). **c** Luciferase assay with E-box-mutated *DISC1* promoter. Luciferase assay was performed in HEK293 cells overexpressing CLOCK and BMAL1 (*n* = 3, biological replicates). Each group was normalized to the respective control (empty vectors were transfected). **d** Luciferase assay conducted using the distal part of *DISC1* promoter with E-box mutations in HEK293 cells overexpressing CLOCK and BMAL1 (*n* = 3, biological replicates). Each group was normalized to the respective control (empty vectors were transfected). **e** Chromatin immunoprecipitation (ChIP) was performed in HEK293 cells transfected with CLOCK or BMAL1 or both to assess direct binding on E-box sequences of *DISC1* promoter (*n* = 3, biological replicates). GFP antibody was utilized to pull down GFP-BMAL1. qPCR was used to quantify the E-box sequences precipitated by GFP-BMAL1. Red arrows indicate estimated primer binding sites for qPCR. For CLOCK and BMAL1 transfection, RFP-CLOCK-myc and GFP-BMAL1 constructs were used for (**a**–**d**), and (**e**). RLA represents relative luciferase activity. Data are means with SEM. *p ≤ 0.05, **p ≤ 0.01, ****p ≤ 0.0001, one-way ANOVA and Tukey’s post-hoc test for (**a**–**c**), (**d**), and (**e**).

Based on the previous characterization of *DISC1* promoter^27^, we further analyzed the region from −982 to +47 bp relative to the transcription start site (TSS). *DISC1* promoter was cloned into pGL3 vector containing luciferase destabilized by PEST sequence^28^. We then assessed the activity of *DISC1* promoter by luciferase assay system. Interestingly, *DISC1* promoter activity was enhanced when CLOCK and BMAL1 were co-expressed, but not when CLOCK was expressed alone (Fig. 2a). Next, fragments of the *DISC1* promoter were sub-cloned into pGL3 vector. The distal part of *DISC1* promoter (-982 to -624 bp relative to TSS) was highly enhanced by CLOCK and BMAL1 co-expression (Fig. 2b). The middle part of *DISC1* promoter (−623 to −324 bp relative to TSS) was also moderately enhanced by CLOCK and BMAL1 transfection, while the proximal part of *DISC1* promoter (−323 to +47 bp relative to TSS) did not show significant enhancement. Therefore, the distal part of *DISC1* promoter is the most responsive region to CLOCK/BMAL1.

To define the binding sites of CLOCK/BMAL1 heterodimer on *DISC1* promoter, we utilized the ‘LASAGNA-Search 2.0’ tool^45,46^. From the prediction based on the previously reported matrix parameter for CLOCK and BMAL1^47^, two candidate binding sites (−718 and −668 bp relative to TSS) were identified (Supplementary Fig. 1). The two predicted sites match with the consensus E-box element, CANNTG, where CLOCK/BMAL1 binds^48–51^. Then, the predicted E-box sequences of *DISC1* promoter were mutated to ‘ACNNTA’ to block their activity. *DISC1* promoter with mutations in either one of the two E-box sequences or both showed lower responses to CLOCK and BMAL1 overexpression than the wild type promoter (Fig. 2c). When the distal part of *DISC1* promoter was tested separately, double mutation of E-box sequences abrogated the response to CLOCK and BMAL1 (Fig. 2d). Then, chromatin immunoprecipitation assay (ChIP) was employed to test whether CLOCK/BMAL1 heterodimer physically binds to the E-box sequences of *DISC1* promoter (Fig. 2e). In CLOCK/BMAL1-cotransfected cells, qPCR signals for both E-boxes (−718 and −668 bp relative to TSS) were markedly increased. In addition, we performed ChIP on mutant E-boxes (−718 and −668 bp relative to TSS) of *DISC1* promoter to examine the binding of CLOCK/BMAL1 heterodimer. The qPCR signals for both E-boxes were significantly decreased (Supplementary Fig. 2). Altogether, the results further support the idea that the two E-box sites within the distal part of *DISC1* promoter are critical for the modulation of *DISC1* expression by CLOCK/BMAL1.

### DISC1 regulates BMAL1 stability and expression of circadian genes

**Fig. 3:**
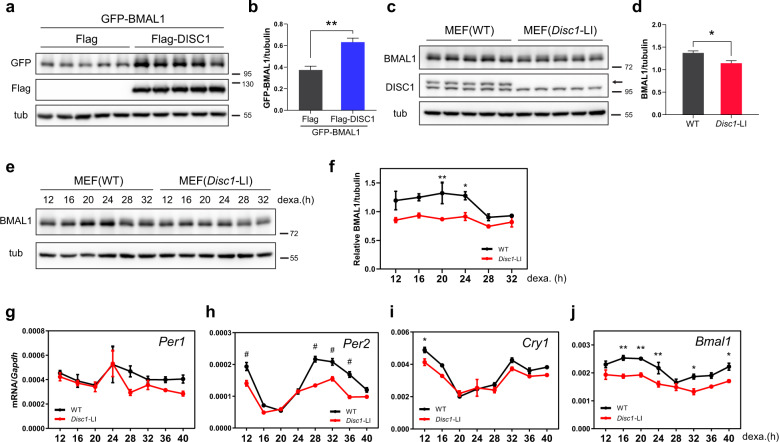
DISC1 regulates BMAL1 stability, and knockout of *Disc1* damps the oscillation of BMAL1. **a** GFP-BMAL1 and Flag-DISC1 were co-transfected into HEK293 cells. Empty Flag vector was used as a control. **b** Quantification of GFP-BMAL1 levels from (**a**) relative to tubulin control (*n* = 5, biological replicates). **c** Endogenous BMAL1 protein expression was decreased in *Disc1* knockout (*Disc1*-LI) MEFs. **d** Quantification of BMAL1 protein levels from (**c**) with tubulin control (*n* = 5, biological replicates). **e** Endogenous BMAL1 protein levels were measured in MEFs following treatment with 1 μM of dexamethasone to synchronize the circadian cycle. **f** Quantification of BMAL1 protein levels from (**e**) relative to tubulin control (*n* = 3 for each time point, biological replicates). The ratios of the relative band intensity at each time point to the band intensities averaged across all time points were presented. **g**–**j** mRNA levels of circadian genes measured by qRT-PCR after dexamethasone synchronization (*n* = 3 for each time point, biological replicates). The expression of circadian genes was reduced in *Disc1* knockout MEFs. The panels show levels of *Per1* (**g**), *Per2* (**h**), *Cry1* (**i**), and *Bmal1* (**j**). Data are means with SEM. *p ≤ 0.05, **p ≤ 0.01, ^#^*p* ≤ 0.0001, unpaired t-test for (**b**, **d**); two-way ANOVA and Sidak’s multiple comparisons for (**f**–**i**), and (**j**).

PERs and CRYs are regulated by CLOCK/BMAL1 through E-box sequences and exhibit oscillatory expression within circadian time^5^. Circadian expression of PERs and CRYs subsequently inhibits CLOCK/BMAL1 via a negative feedback loop. Since *DISC1* expression is regulated by CLOCK/BMAL1 through E-box sequences and displays an oscillatory expression, we hypothesized that DISC1 protein may also affect the molecular circadian system, and we scrutinized the potential links. Interestingly, we observed that ectopic expression of DISC1 enhanced BMAL1 protein level in HEK293 cells (Fig. 3a, b). Conversely, endogenous BMAL1 protein level was decreased in mouse embryonic fibroblasts (MEFs) derived from *Disc1*-LI mice, a *Disc1*-deficient mouse line^24^ (Fig. 3c, d).

To verify whether DISC1 deficiency affects BMAL1 oscillation over circadian time, we analyzed BMAL1 protein expression at each circadian time after dexamethasone treatment (Fig. 3e, f). The circadian oscillation of BMAL1 was significantly damped in *Disc1*-LI MEFs, and this effect was higher when BMAL1 levels were at their peak, namely 20 and 24 h after dexamethasone treatment. Moreover, the mRNA levels of circadian genes under the control of BMAL1 were concomitantly reduced in *Disc1*-LI MEFs (Fig. 3g–j). Given that the knockout of *Disc1* damps BMAL1, thereby also decreasing the mRNA expression of *Per2*, *Cry1*, and *Bmal1*, these data strongly suggest that DISC1 may function as a stabilizer of BMAL1.

### *Disc1* knockout mice display altered circadian behavior

**Fig. 4:**
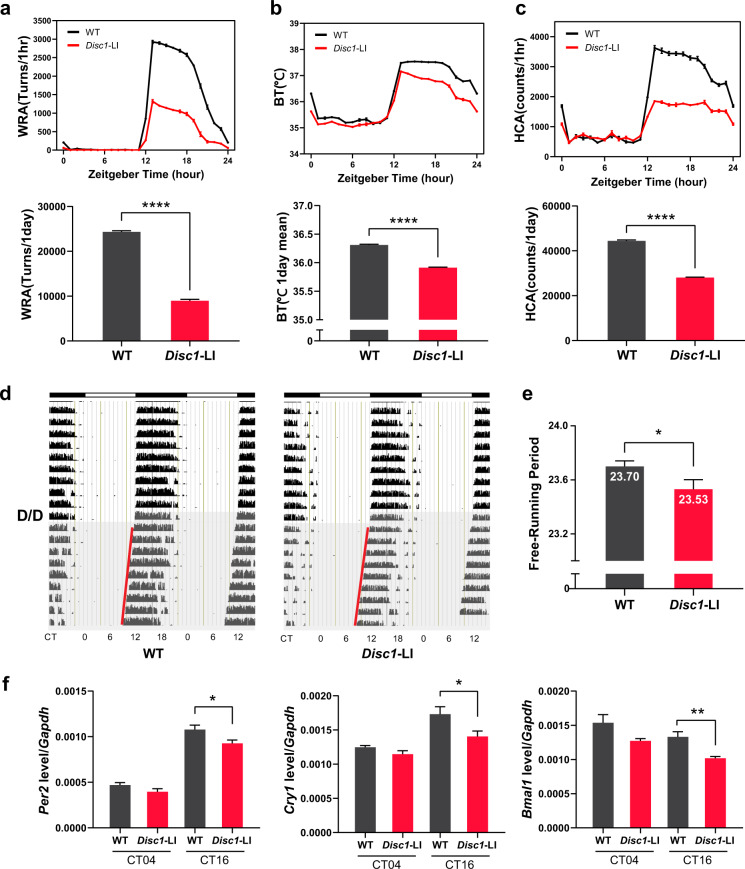
*Disc1* knockout mice exhibit altered circadian behaviors. **a**–**c** Overall circadian behaviors during L/D cycle (*n* = 10, biological replicates). Mice were fully entrained to the light/dark (L/D; 12-h/12-h) cycle for at least two weeks. For the next 10 days with the same lighting schedule, light/dark profiles were generated as described in “Methods”. Then, mice were released to a dark/dark (D/D; constant dark) cycle for another 10 days to determine the internal circadian period. **a** WRA, wheel-running activity; **b** BT, body temperature; **c** HCA, home cage activity. Compared to wild type mice, *Disc1* knockout mice (*Disc1*-LI) showed reduced values during the nighttime. Total measured values per one day were depicted as bar graphs for wheel-running and home cage activities. For body temperature, averaged values were used. **d**, **e** Free-running periods were measured for wild type (*n* = 16, biological replicates) and *Disc1* knockout (*n* = 10, biological replicates) mice under the D/D cycle. *Disc1* knockout mice showed a shorter Free-running period than wild type mice. **f** mRNA levels of circadian genes at circadian time of 4 h (CT04) and 16 h (CT16) (*n* = 4 for 4 h, *n* = 5 for 16 h, biological replicates). Hippocampus was dissected from wild type or *Disc1* knockout mice. Data are means with SEM. *p ≤ 0.05, **p ≤ 0.01, ****p ≤ 0.0001, unpaired *t*-test for (**a**), (**b**), (**e**) and (**f**); unpaired *t*-test with Welch’s correction for (**c**).

Since *Disc1* knockout decreases the expression of circadian genes and decreases protein levels of BMAL1, we hypothesized that *Disc1* knockout would also affect circadian behaviors. To test this hypothesis, we conducted circadian behavioral tests with *Disc1*-LI mice. We first entrained mice to a 12-h light and 12-h dark cycle prior to the observation of light/dark profile of circadian behavior. Notably, *Disc1*-LI mice exhibited lowered amplitude of circadian behaviors: wheel-running activity (Fig. 4a), body temperature (Fig. 4b), and home cage activity (Fig. 4c). We then monitored the wheel-running activity profile in a constant dark/dark cycle to observe the internal circadian period of mice, which is represented by the free-running period (Fig. 4d, e). *Disc1*-LI mice displayed a shorter free-running period than wild type mice. Furthermore, we examined phase perturbations from external light cues (Supplementary Fig. 3). We gave 30 min of the light pulse to mice at circadian time 14 h or 20 h for phase delay assay or phase advance assay, respectively. *Disc1*-LI mice exhibited a tendency toward an increase in phase delay and phase advance experiments. However, both did not reach statistical significance.

We also checked the expression of circadian genes at the transcript level. Consistent with the MEFs data, at circadian time 16 h, *Disc1*-LI mice showed lower expression of *Per2*, *Cry1*, and *Bmal1* in the hippocampus than wild type mice (Fig. 4f). These results further demonstrate that DISC1 has a modulatory role for circadian rhythm likely through stabilizing BMAL1, a core component of the molecular clock.

### DISC1 regulates the stability of BMAL1 by modulating its ubiquitination through GSK3β

**Fig. 5:**
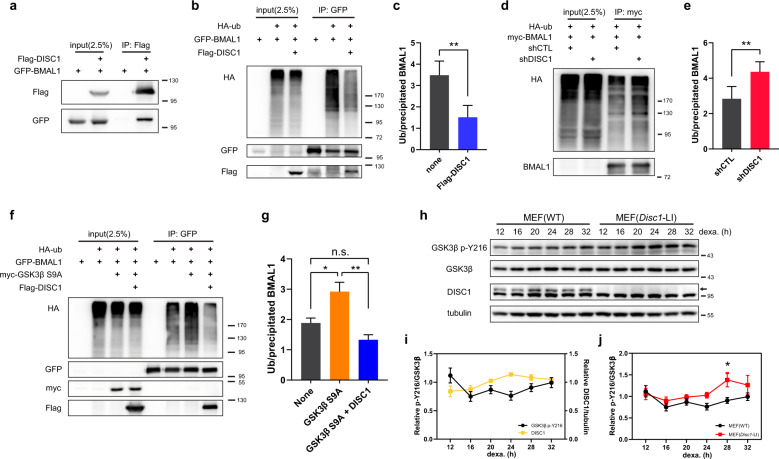
DISC1 inhibits BMAL1 ubiquitination in association with suppression of Y216 phosphorylation of GSK3β. **a** Co-immunoprecipitation of DISC1 and BMAL1. Flag-DISC1 and GFP-BMAL1 were transfected in HEK293 cells and precipitated with Flag-DISC1. **b** Ubiquitination assay for BMAL1. HA-ubiquitin was transfected in HEK293 cells to assess the ubiquitination of BMAL1. GFP-BMAL1 was transfected with or without Flag-DISC1. **c** Quantification of ubiquitin level from (**b**) relative to precipitated BMAL1 level (*n* = 5, biological replicates). **d** Ubiquitination assay of BMAL1 with DISC1 knockdown. HA-ubiquitin was transfected in HEK293 cells to assess the ubiquitination of BMAL1. myc-BMAL1 was transfected with shCTL or shDISC1 for knockdown of DISC1. **e** Quantification of ubiquitin level from (**d**) relative to precipitated BMAL1 level (*n* = 4, biological replicates). **f** Ubiquitination assay of BMAL1 with GSK3β S9A and DISC1. HA-ubiquitin was transfected in HEK293 cells to assess the ubiquitination of BMAL1. GFP-BMAL1 was transfected with myc-GSK3β S9A and Flag-DISC1 according to each combination of constructs. **g** Quantification of ubiquitin level from (**f**) relative to precipitated BMAL1 level (*n* = 4, biological replicates). **h** Western blot analysis of Y216 phosphorylation of GSK3β in wild type and *Disc1* knockout (*Disc1*-LI) MEFs. MEFs were treated with 1 μM of dexamethasone to synchronize the circadian cycle. Western blot of GSK3β was conducted in different gel from others to avoid re-probing interference from previous band blot (p-Y216 GSK3β). Western blot was conducted in parallel with the same amount of identical samples. **i** Quantification of DISC1 and Y216 phosphorylation of GSK3β from (**h**) (*n* = 6, *n* = 3 for DISC1, biological replicates). Relative intensities of DISC1 and p-Y216 GSK3β relative to tubulin and total GSK3β, respectively, were subjected to analyses. The ratios of the relative band intensity at each time point to the band intensities averaged across all time points were presented. **j** Quantification of Y216 phosphorylation levels in wild type and *Disc1* knockout MEFs from (**h**) (*n* = 6, biological replicates). The intensity of p-Y216 GSK3β relative to total GSK3β was subjected to analyses. The ratios of the relative band intensity at each time point to the band intensities averaged across all time points were presented. Data are means with SEM. *p ≤ 0.05, **p ≤ 0.01, paired *t*-test for (**c**, **e**), one-way ANOVA and Tukey’s post-hoc test for (**g**), two-way ANOVA and Sidak’s multiple comparisons for (**j**).

Next, we sought to elucidate how DISC1 regulates the stability of BMAL1. Because DISC1 is known to function as a scaffold protein, we examined the interaction between DISC1 and BMAL1. DISC1 interacted with BMAL1 as shown by co-immunoprecipitation with overexpressed DISC1 and BMAL1 (Fig. 5a). We further confirmed the co-immunoprecipitation in MEFs and mouse brain lysates (Supplementary Fig. 4), suggesting that DISC1 may directly affect BMAL1 function.

Ubiquitination is widely known as a signal for protein degradation^52^. Hence, we conducted ubiquitination assays and observed that ubiquitination of BMAL1 was inhibited upon co-expression of DISC1 (Fig. 5b, c), consistent with the finding that DISC1 increases BMAL1 levels. Conversely, ubiquitination of BMAL1 was enhanced in *DISC1* knockdown cells (Fig. 5d, e). Thus, we concluded that DISC1 binds to BMAL1 and inhibits its ubiquitination.

The phosphorylation of glycogen synthase kinase 3β (GSK3β) Ser9 is inhibitory, while Tyr216 phosphorylation enhances its activity^53^. DISC1 can inhibit the Tyr216 phosphorylation (p-Y216) of GSK3β to impede its enzymatic activity^30^. On the other hand, GSK3β regulates multiple circadian proteins^54^, such as CRY2^55,56^, CLOCK^57^, BMAL1^11^, and REV-ERBα^58^, by modulating their stability^10^. Particularly, BMAL1 phosphorylation by GSK3β promotes its ubiquitination^11^. In addition, as shown in previous reports^11,30^, GSK3β interacts with DISC1 and BMAL1 in co-immunoprecipitation experiments (Supplementary Fig. 4b). Thus, we tested if DISC1 contributes to the regulation of BMAL1 ubiquitination through GSK3β. An activity-enhanced form of GSK3β by mutation of Ser9 to Ala increased the ubiquitination of BMAL1 as previously reported (Fig. 5f, g). However, this effect was abolished upon co-expression with DISC1.

To determine the effect of DISC1 on GSK3β over circadian time, we examined the level of p-Y216 in MEFs after dexamethasone synchronization (Fig. 5h). When DISC1 was at its peak, 24 h after synchronization, p-Y216 level was low (Fig. 5i). Subsequently, p-Y216 level gradually increased, while DISC1 level decreased. Moreover, p-Y216 level in *Disc1*-LI MEFs was increased at 28 h after dexamethasone treatment compared to wild type (Fig. 5j). Taken together, these results indicate that DISC1 suppresses p-Y216 of GSK3β according to circadian time, thereby enhancing BMAL1 stability.

## Discussion

In this study, we showed that DISC1 has novel functions on the molecular circadian clock, further expanding the repertoire of the diverse roles of DISC1 in various cell types. Interestingly, the functional interaction between DISC1 and the molecular clock is reciprocal; DISC1 modulates the quality of the molecular clock by regulating the GSK3β-mediated BMAL1 stability and, at the same time, *DISC1* expression itself is under control of CLOCK/BMAL1, displaying an oscillating expression pattern according to circadian time (Supplementary Fig. 5). The oscillating level of DISC1 is supposed to affect the oscillating level of BMAL1 protein, which will, in turn, affect the *DISC1* promoter activity. This regulatory loop is achieved by the presence of E-box elements in the *DISC1* promoter, which are targets of CLOCK/BMAL1 heterodimer. We identified the two major E-boxes controlled by BMAL1/CLOCK, but more cis-regulatory elements participating in the circadian expression of *DISC1* directly or indirectly may still exist. This notion is supported by the observation that the *DISC1* promoter activity was not totally abolished by the mutations of both E-box elements. Presumably, as a result, DISC1 deficiency leads to damped expressions of circadian genes. The impacts on the circadian genes were quantitatively differential (Fig. 3g–j), likely due to the heterogeneity of the compositions and their collective reactivity of the cis-regulatory elements responsible for circadian control^59,60^. Further clarification of this novel modulatory pathway will establish a complete view of the molecular clock.

The peak of DISC1 is similar to the peak of BMAL1, occurring ~20–24 h after dexamethasone treatment (Figs. 3f and 5i). Moreover, the expression pattern of DISC1 in the brain (Fig. 1b, c, e) is also consistent with the previously reported expression pattern of BMAL1^9,61,62^, supporting a protective role of DISC1 for BMAL1. In this regard, the shorter period seen in the *Disc1*-LI mouse appears counterintuitive, given that mice with low BMAL1 show a more extended period^63,64^ and *Bmal1* knockout mice exhibit arrhythmia^65,66^. Notably, however, DISC1 functionally down-regulates GSK3β by inhibiting p-Y216. Although there are conflicting results on the relationship between GSK3β and circadian period^67,68^, GSK3β gain-of-function tends to shorten the circadian period^69,70^. Thus, it is possible that the shortened period in *Disc1*-LI mice is due to the enhanced activity of GSK3β, which is consistent with the notion that DISC1 prevents BMAL1 from premature degradation to maintain the robustness of circadian rhythmicity.

We demonstrated that DISC1 expression oscillates according to circadian time. This finding suggests that the previously known functions of DISC1, from embryonic neurodevelopment to adult neurogenesis, may also be regulated by the circadian system. In this respect, the oscillating phosphorylation of Y216, a regulatory hotspot of GSK3β activity controlled by DISC1^30^, is noteworthy. The phosphorylation of Y216 is critical for neural progenitor proliferation, which may indicate that DISC1-related neurodevelopmental processes, including neuronal signaling, neuronal morphogenesis, and spine regulation^17^, are also under circadian control. Moreover, DISC1 downregulation augments adult neurogenesis in the hippocampus^71,72^ in conjunction with memory formation^73^. Indeed, memory formation and adult neurogenesis are circadian-related phenomena^74,75^. For example, the number of BrdU-positive cells in the hippocampal subgranular zone changes over circadian time, with relatively impaired neurogenesis from late night to early daytime (peak neurogenesis at 15 h)^75^, which corresponds to the level of DISC1 protein expression in the hippocampus shown in this work (Fig. 1e). Therefore, it will be interesting to test whether DISC1 acts as an output pathway that links the molecular clock and neurodevelopmental program.

A natural deletion of *Disc1*, resulting in truncation after amino acid residue 528, did not alter the sleep phenotypes^76^, which is counterintuitive to our results from *Disc1*-LI mice. However, it is noteworthy that the truncated DISC1 still retains the region, amino acid residues 195-228, for the inhibition of GSK3β activity^30^, which is a crucial step of the regulation of BMAL1 stability by DISC1 according to the observations in this study. Its more robust circadian phenotype shown in this study is likely to be a reflection of complete *Disc1* loss-of-function. Anyhow, the involvement of DISC1 in the regulation of the molecular circadian clock is intriguing in that sleep and circadian rhythm disruption (SCRD) is a common feature of various psychiatric disorders^77^. Components of molecular clocks were proposed as factors of SCRD^78^, though the precise molecular mechanisms underlying this phenomenon are still elusive. Although the genetic association of DISC1 with the various psychiatric conditions is still to be further clarified, the identification of DISC1 as a novel regulator of the molecular clock may suggest its role in the interface between some aspects of psychiatric disorders and the circadian system. Notably, rhythmic expression of genes, including *PER2* and *CRY2*, is abolished in a group of patients with schizophrenia^79^; similarly, expression of *PER2* and *CRY1* is impaired in some schizophrenia patients^80^. In our study, an interesting correlation was observed in the *Disc1* knockout mouse, where the expression of the circadian genes was also diminished. Thus, although the nature of the correlation needs further validation, the roles of DISC1 in the molecular clock may provide a novel mechanistic insight into the molecular links between SCRD and psychiatric disorders.

## Supplementary information

Supplementary Figure 1

Supplementary Figure 2

Supplementary Figure 3

Supplementary Figure 4

Supplementary Figure 5

## Supplementary information

**Supplementary information** The online version contains supplementary material available at 10.1038/s41398-021-01212-1.
