# Supplementary material for: Disrupted-in-schizophrenia 1 enhances the quality of circadian rhythm by stabilizing BMAL1
Source: Transl Psychiatry. 2021 Feb 4;11:110. doi: 10.1038/s41398-021-01212-1 (PMC7862247; doi:10.1038/s41398-021-01212-1)
Supplement: Supplementary file 1 — Supplementary Figure 1 [file 41398_2021_1212_MOESM1_ESM.pdf]

a

Name	Sequence	Position (0-base)	Strand	Score	p-value	E-value
CLOCK-BMAL1	AGAGCACGTGCATAG	259	-	11.65	0	0
CLOCK-BMAL1	AGCTCATGTGTAGCC	310	+	10.02	0	0
CLOCK-BMAL1	AGAGCACGTGCATA	260	-	8.84	0	0
CLOCK-BMAL1	GCTACACATGAGCT	310	-	6.13	0.0005	0.51

b

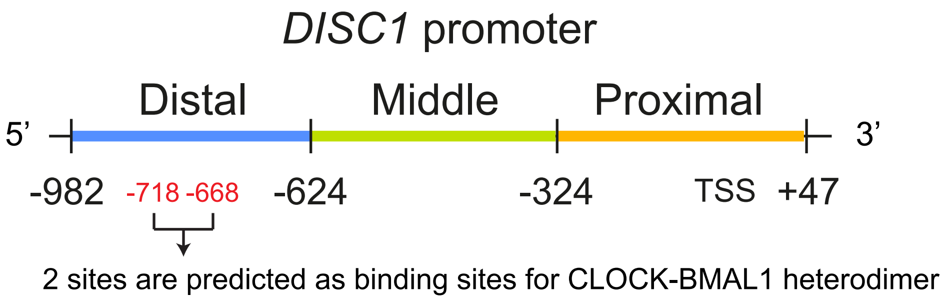

Supplementary Figure 1. CLOCK and BMAL1 binding site prediction on *DISC1* promoter by LASAGNA-Search 2.0 tool.
a A result from LASAGNA-Search 2.0 tool. **b** Two sites (-718 and -668 bp relative to TSS) were predicted as binding sites for CLOCK and BMAL1 heterodimer.
