# Supplementary material for: Disrupted-in-schizophrenia 1 enhances the quality of circadian rhythm by stabilizing BMAL1
Source: Transl Psychiatry. 2021 Feb 4;11:110. doi: 10.1038/s41398-021-01212-1 (PMC7862247; doi:10.1038/s41398-021-01212-1)
Supplement: Supplementary file 2 — Supplementary Figure 2 [file 41398_2021_1212_MOESM2_ESM.pdf]

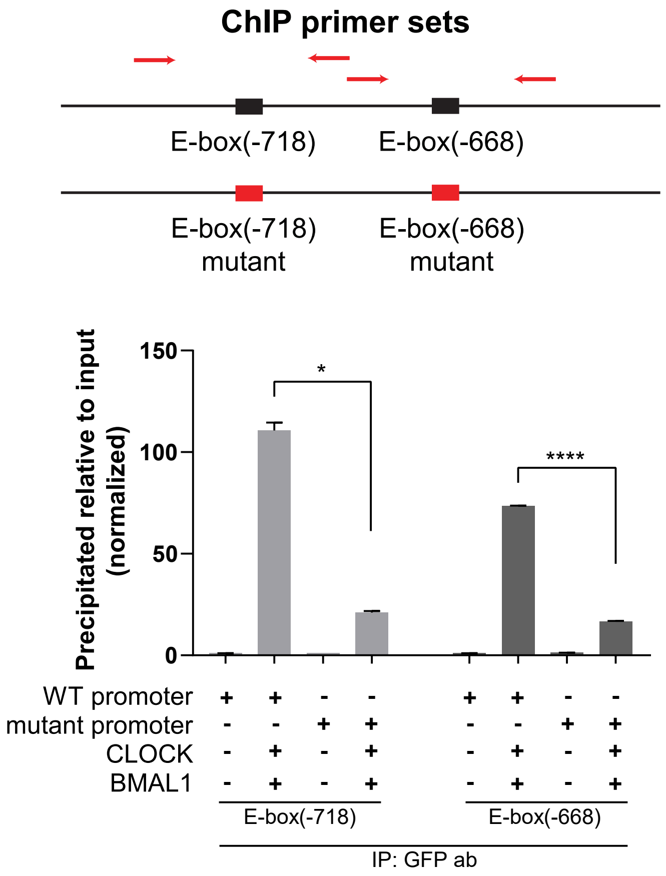

Supplementary Figure 2. Chromatin immunoprecipitation with E-box mutations on *DISC1* promoter.

Chromatin immunoprecipitation was performed with wild type or E-box mutated *hDISC1* promoter constructs (*n* = 3, biological replicates). *hDISC1* promoter constructs were transfected in HEK293 cells with CLOCK and BMAL1. Wild type (WT) *hDISC1* promoter construct and E-box (-718, -668) mutated *hDISC1* promoter construct with pGL3 vector were utilized. RFP-CLOCK-myc and GFP-BMAL1 constructs were used. GFP antibody was utilized to pull down GFP-BMAL1. qPCR was used to quantify the E-box sequences precipitated by GFP-BMAL1. Red arrows indicate estimated primer binding sites for qPCR. Data are means with SEM. *p≤0.05, ****p≤0.0001, Welch's ANOVA, Dunnett T3 test.
