# Supplementary material for: Disrupted-in-schizophrenia 1 enhances the quality of circadian rhythm by stabilizing BMAL1
Source: Transl Psychiatry. 2021 Feb 4;11:110. doi: 10.1038/s41398-021-01212-1 (PMC7862247; doi:10.1038/s41398-021-01212-1)
Supplement: Supplementary file 3 — Supplementary Figure 3 [file 41398_2021_1212_MOESM3_ESM.pdf]

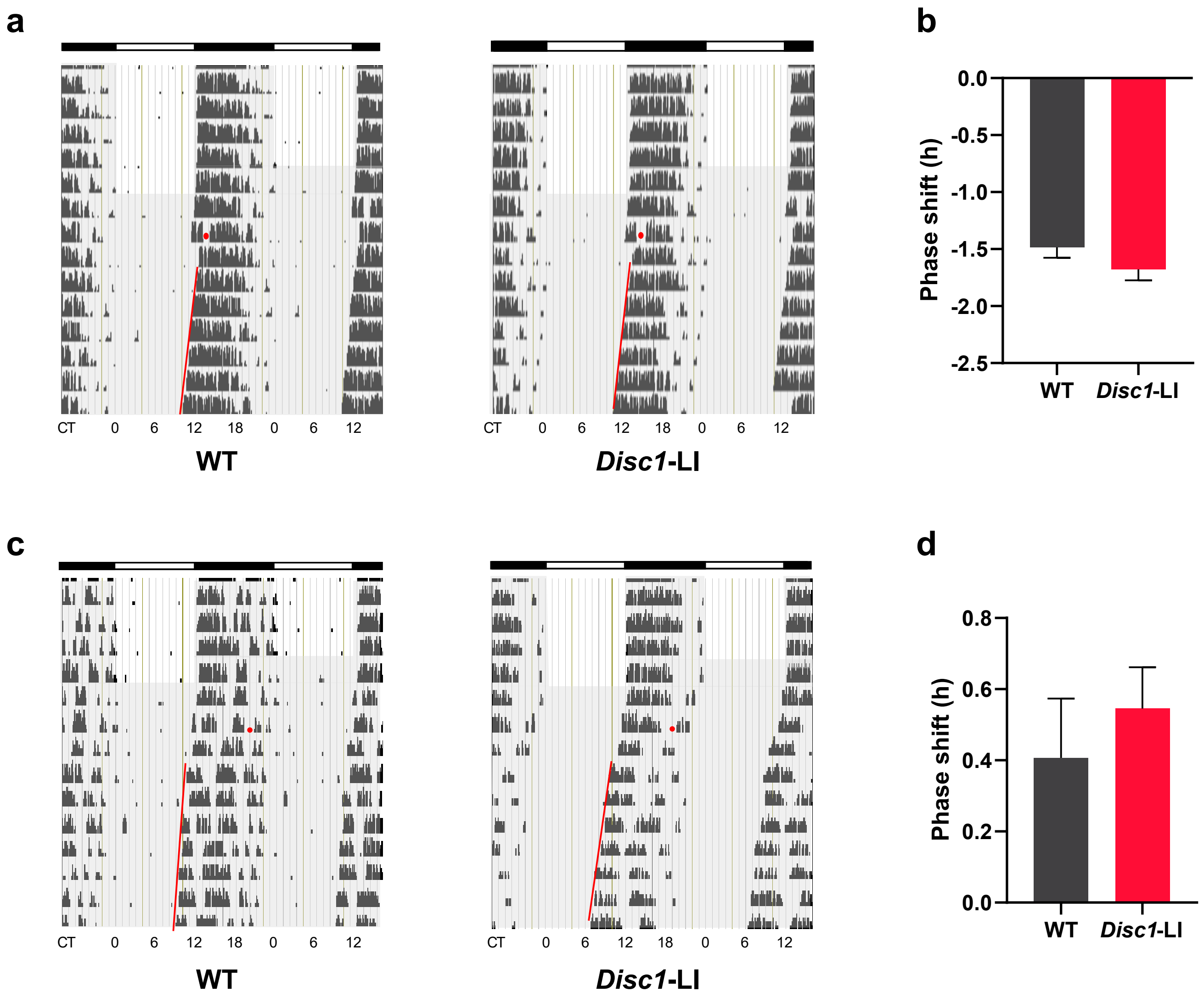

Supplementary Figure 3. Phase delay and phase advance assay with *Disc1*-LI mice. **a**, **b** Phase delay experiment with 30 min of light pulse at circadian time 14hr. Wild type (WT) mice and *Disc1* knockout (*Disc1*-LI) mice were utilized (n = 11 for WT, n = 8 for *Disc1*-LI, biological replicates). **c**, **d** Phase advance experiment with 30 min of light pulse at circadian time 20hr. Wild type mice and *Disc1* knockout (*Disc1*-LI) mice were utilized (n = 11 for WT, n = 8 for *Disc1*-LI, biological replicates). Data are means with SEM.
