# Supplementary material for: Disrupted-in-schizophrenia 1 enhances the quality of circadian rhythm by stabilizing BMAL1
Source: Transl Psychiatry. 2021 Feb 4;11:110. doi: 10.1038/s41398-021-01212-1 (PMC7862247; doi:10.1038/s41398-021-01212-1)
Supplement: Supplementary file 4 — Supplementary Figure 4 [file 41398_2021_1212_MOESM4_ESM.pdf]

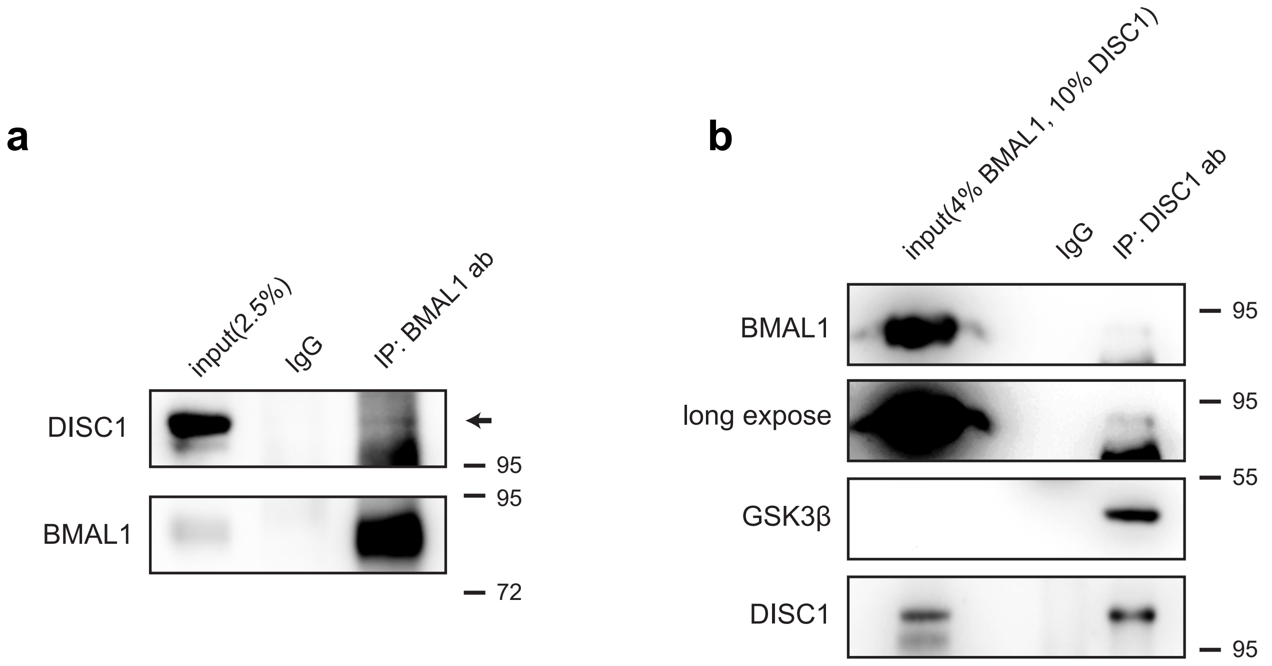

Supplementary Figure 4. Endogenous co-immunoprecipitation of DISC1 and BMAL1.

a Endogenous co-immunoprecipitation of DISC1 and BMAL1 in MEFs. BMAL1 antibody was utilized for the precipitation. **b** Endogenous co-immunoprecipitation of DISC1, BMAL1 and GSK3β in mouse whole brain lysate. DISC1 antibody was utilized for the precipitation. Longer expose of BMAL1 blot was also included.
