# Supplementary material for: Disrupted-in-schizophrenia 1 enhances the quality of circadian rhythm by stabilizing BMAL1
Source: Transl Psychiatry. 2021 Feb 4;11:110. doi: 10.1038/s41398-021-01212-1 (PMC7862247; doi:10.1038/s41398-021-01212-1)
Supplement: Supplementary file 5 — Supplementary Figure 5 [file 41398_2021_1212_MOESM5_ESM.pdf]

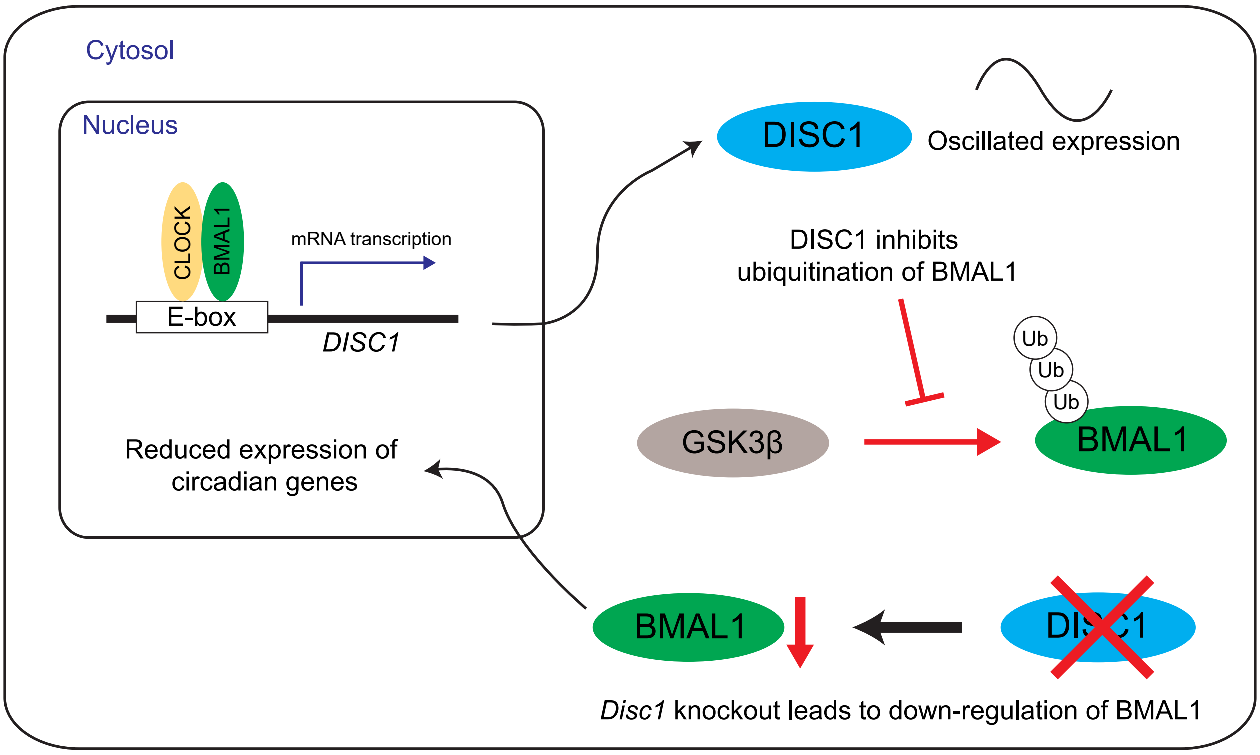

Supplementary Figure 5. Roles of DISC1 in molecular circadian system.
DISC1 is regulated by CLOCK/BMAL1 heterodimer and exhibits oscillating expression. Importantly, DISC1 stabilizes BMAL1 by inhibiting its ubiquitination. DISC1 deficiency leads to down-regulation of BMAL1 and other circadian genes, thereby damping the circadian system.
